# Antioxidant Potential and Antibacterial Activities of Caucasian Endemic Plants *Sempervivum transcaucasicum* and *Paeonia daurica* subsp. *mlokosewitschii* Extracts and Molecular In Silico Mechanism Insights

**DOI:** 10.3390/jox15040109

**Published:** 2025-07-04

**Authors:** Valentina Mittova, Marina Pirtskhalava, Zurab R. Tsetskhladze, Khatuna Makalatia, Alexander Loladze, Irakli Bebiashvili, Tinatin Barblishvili, Ana Gogoladze, Giovanni N. Roviello

**Affiliations:** 1 Scientific-Research Institute of Experimental and Clinical Medicine, University Geomedi, King Solomon II Str. 4, 0114 Tbilisi, Georgia; zurab.tsetskhladze@geomedi.edu.ge (Z.R.T.); khatuna.makalatia@geomedi.edu.ge (K.M.); 2 Faculty of Medicine, University Geomedi, King Solomon II Str. 4, 0114 Tbilisi, Georgia; marina.pirtskhalava@geomedi.edu.ge (M.P.); aleksandre.loladze@geomedi.edu.ge (A.L.); irakli.bebiashvili@geomedi.edu.ge (I.B.); 3 National Botanical Garden of Georgia, Botanikuri Street 1, 0105 Tbilisi, Georgia; tinabar7@yahoo.com (T.B.); agogoladze@yahoo.com (A.G.); 4 Institute of Biostructures and Bioimaging, Italian National Council for Research (IBB-CNR), Area di Ricerca Site and Headquarters, Via Pietro Castellino 111, 80131 Naples, Italy

**Keywords:** Caucasian endemic plants, *Sempervivum transcaucasicum*, *Paeonia daurica* subsp. *mlokosewitschii*, antioxidant activity, antibacterial activity, in silico docking, OmpX protein, *Escherichia coli*

## Abstract

Antioxidants derived from plant extracts have attracted considerable attention due to their potential in mitigating oxidative damage through free radical scavenging mechanisms. Although 700 species have been used for centuries in Georgian traditional medicine, the chemical composition and antioxidant and antibacterial properties of Caucasian endemic medicinal plants remain largely unknown. In this study, the antioxidant and antibacterial activities of leaf and root extracts of Caucasian endemic medicinal plants *Sempervivum transcaucasicum* Muirhead and *Paeonia daurica* subsp. *mlokosewitschii* (Lomakin) D. Y. Hong were investigated. The highest antioxidant activity and phenolic and flavonoid content were revealed in *Paeonia daurica* leaf extract. The analysis of the content of water-soluble antioxidants revealed the highest content of reduced glutathione and ascorbate in *Paeonia daurica* leaves. Moreover, the antibacterial activity of leaf and root extracts against *Escherichia coli* ATCC 25922 strain was investigated, and minimal inhibitory concentration (MIC) values were determined. While the antibacterial activity against *E. coli* ATCC 25922 was not revealed for the *Sempervivum transcaucasicum* leaf extract, antibacterial properties were detected for the root extract (MIC 5 mg/mL). Collectively, the highest antibacterial activity was revealed for *Paeonia daurica* leaf and root extracts (MIC 2 mg/mL and 3 mg/mL, respectively). From a molecular perspective, molecular docking simulations were performed using HDOCK software, with reduced glutathione and ascorbic acid as ligands, in order to analyse their potential binding affinity to the OmpX protein. Inhibiting this protein would likely disrupt bacterial function and produce an antibacterial effect. Our results provide a possible mechanism for the antibacterial activity of *Paeonia daurica* subsp. *mlokosewitschii*. Overall, the results of the study demonstrate the potential of Caucasian endemic medicinal plants as natural antioxidants and antimicrobial agents.

## 1. Introduction

Heavy metals, including cadmium, lead, and mercury, are notorious for their detrimental effects on human health, primarily due to their ability to induce oxidative stress [1,2,3]. These toxic elements generate reactive oxygen species (ROS): superoxide radicals (O_2_^•−^), hydrogen peroxide (H_2_O_2_), hydroxyl radicals (•OH), and singlet oxygen (^1^O_2_) [4]. At low concentrations, ROS are normally produced in the cell and play several beneficial roles for the organism, such as involvement in the synthesis of some cellular structures and the host defence system to fight pathogens [5]. However, an imbalance between the production of ROS and their elimination causes oxidative stress, negatively affecting membranes, lipids, proteins, lipoproteins, and DNA [6]. Over time, this oxidative burden contributes to a range of severe health complications, including neurodegenerative disorders [7], cardiovascular diseases [8], and even cancer [9]. Given the widespread exposure to heavy metal pollutants through contaminated air, water, and food sources, addressing this issue has become increasingly urgent.

Plants are phytochemical hubs, containing a variety of both water-soluble antioxidants, such as ascorbate (ASC) and glutathione (GSH) and secondary metabolites, polyphenols, flavonoids and terpenoids, which participate in the ROS detoxification [2,10]. In recent years, natural antioxidants derived from plant extracts have gained considerable attention as potential protective and therapeutic agents against heavy-metal-induced oxidative damage [11]. Rich in bioactive compounds such as flavonoids, phenolic acids, and carotenoids [12], these plant-based remedies exhibit powerful free-radical-scavenging properties that can mitigate oxidative harm, support cellular defence mechanisms, and enhance overall resilience to environmental toxins. The exploration of medicinal plants for their antioxidant efficacy not only underscores their therapeutic promise but also highlights their potential role in future preventive strategies for reducing the long-term impact of heavy metal exposure on human health.

The great variety of climate zones and landscapes makes Georgian nature renowned for its unique woodland habitats and immense biodiversity. Georgia harbours a variety of plants with high antioxidant potential, many of which are endemic (reviewed in [13]). Around 700 plant species are used in Georgian traditional medicine, and a plethora of evidence validates that plant biomedical applications play a role in the prevention and treatment of cardiovascular, inflammatory and neurodegenerative disorders; cancer; and diabetes (reviewed in [13,14]). However, the chemical composition, antioxidant activity, and antibacterial properties of Caucasian endemic medicinal plants are mainly unexplored.

*Sempervivum transcaucasicum* Muirhead is a succulent perennial with stems densely covered with glandular hairs and rosette leaves [15]. The plant grows in the Kartli and Meskhet-Javakheti regions of Georgia, Armenia, Azerbaijan, and the NE of Turkey [16,17,18], representative of the genus Sempervivum (47 species, 16 heterotypic infraspecific taxa, 16 natural hybrids) [19]. In traditional medicine, the 96% ethanol tincture of the aboveground part of the species or freshly detached homogenised leaves are used as spasmolytic, antiseptic [18], and antibacterial remedies [20]. The phytochemical characterisation of this species was not performed, but the high content of phenolic compounds and antioxidant and antibacterial activity were shown for representatives of the genus Sempervivum [21,22].

*Paeonia daurica* subsp. *mlokosewitschii* (Lomakin) D. Y. Hong is a perennial herbaceous plant, with biternate, greyish or greyish-green leaves; widely open yellowish flowers; a lignified, branched rhizome; and spindle-shaped roots [23]. The species is endemic to the Caucasus, distributed in the North Caucasus and Transcaucasus [16]. Nowadays, it occurs only in two regions of Georgia: Kakheti (Lagodekhi) and Kiziki (Shiraki) [24]. The plant is representative of the genus Paeonia, including 33 species and 26 subspecies worldwide [25]. Leaves and roots of *Paeonia daurica* subsp. *mlokosewitschii* (Lomakin) D. Y. Hong possess antioxidant, analgesic, anti-inflammatory, antibacterial, antitumor, antispasmodic, hypotensive, and neuroprotective properties [26,27,28,29], and 96% or 40% ethanol tincture of leaves or roots of this species have been used in traditional medicine for curing tuberculosis, bronchitis, pneumonia, kidney diseases, stomach ache, infectious hepatitis, convulsions, and diseases of the central nervous system [24]. The phytochemical composition of *Paeonia daurica* subsp. *mlokosewitschii* (Lomakin) D. Y. Hong was not investigated; however, compounds isolated from different species of the genus Paeonia include 153 monoterpenoid glucosides, 59 flavonoids, 53 tannins, 15 stilbenes, 49 triterpenoids and steroids, 61 phenols, and 61 other compounds (reviewed in [25]).

Given the documented use of *Sempervivum transcaucasicum* and *Paeonia daurica* subsp. *mlokosewitschii* extracts in traditional medicine [18,24], these plants could be explored as potential sources of bioactive compounds for food supplements. Interestingly, extracts of the Paeonia genus are widely used in traditional medicine across various countries, particularly in Asia, supporting their established ethnopharmacological relevance [30]. On the other hand, *Sempervivum transcaucasicum* is documented among the edible plants traditionally gathered and used by Yezidis and Kurds in Armenia, indicating its local ethnobotanical use as a food source [31].

Their antioxidant properties may contribute to protective mechanisms against xenobiotic-induced oxidative damage, caused by reactive oxygen species and reactive nitrogen species. Various classes of xenobiotics, such as pesticides, pharmaceutical compounds, personal care products, illicit drugs, industrial products, and nuclear waste, were shown to induce oxidative damage [2]. The antibacterial activity of Georgian medicinal plants suggests potential applications in supporting microbial balance and overall health.

This study aimed to investigate the antioxidant activity, as well as the total content of phenols and flavonoids, and the content of the most abundant water-soluble antioxidants (ascorbate and glutathione) in two species, *Sempervivum transcaucasicum* Murihead (family Crassulaceae) and *Paeonia daurica* subsp. *mlokosewitschii* (Lomakin) D. Y. Hong (family Paeoniaceae), used in traditional Georgian medicine. Based on the traditional use of studied medicinal plants, we proposed that leaf and root extracts of these species exhibit antibacterial activity. To test this hypothesis, this study also aimed to evaluate the antibacterial activity of leaf and root extracts of these plants against *Escherichia coli*, one of the most frequent causes of many bacterial infections.

## 2. Materials and Methods

The materials used in this study included aluminium chloride hexahydrate, ammonium molybdate, amoxicillin, ascorbic acid, dimethyl sulfoxide (DMSO), 5,5′-dithiobis(2-nitrobenzoic acid) (DTNB), 2,2-diphenyl-1-picrylhydrazyl (DPPH), dithiothreitol (DTT), *N*-ethylmaleimide, Folin–Ciocalteu’s phenol reagent, gallic acid, glutathione, glutathione reductase from *S. cerevisiae,* methanol, NADPH, rutin, sodium carbonate, sodium nitrite, sodium phosphate, and 2-vinylpyridine purchased from Sigma Aldrich (St. Louis, MA, USA). The LB broth and LB agar plates for *E. coli* were purchased from LLC Elavia Biopreparations, Tbilisi, Georgia.

### 2.1. Plant Material

Plants were collected on the same day in July 2024 in the National Botanical Garden of Georgia (Tbilisi). The geographic coordinates where the plant material was collected were as follows: *Sempervivum transcaucasicum* Murihead (N41.68599° E044.80275°) and *Paeonia daurica* subsp. *mlokosewitschii* (Lomakin) D. Y. Hong (N41.68574° E044.80217°). *Paeonia daurica* subsp. *mlokosewitschii* (Lomakin) D. Y. Hong mature generative plants were collected after the end of the flowering period, in the phase of seed maturation. *Sempervivum transcaucasicum* Murihead plants were collected before the flowering period. The harvest time was chosen according to Georgian traditional medicine: *Sempervivum transcaucasicum* Murihead plants are usually collected before flowering, and *Paeonia daurica* subsp. *mlokosewitschii* (Lomakin) D. Y. Hong plants are collected after flowering.

Five plants of each species were collected, placed in paper bags, and transported to the laboratory within 1 h. For *Sempervivum transcaucasicum,* leaves were separated from the rosette and roots and collected from each plant. For *Paeonia daurica* subsp. *mlokosewitschii*, fully developed leaves of the middle part of the stem and roots were collected from each plant. Samples of fresh plant material were frozen in liquid nitrogen and stored at −80 °C.

### 2.2. Drying Processes

The leaves and roots of both plant species (5 g) were freeze-dried using a DW-10N freeze dryer (Drawell, China) in a 500 mL vacuum flask at 10 Pa and a final condenser temperature of −55 °C, until the plant material reached a constant weight, as was determined by measuring the dry weight.

### 2.3. Extraction of Plant Samples for the Determination of Antioxidant Activity (DPPH Test and TAC Methods) and Total Phenolic and Flavonoid Content

The extraction efficiencies of different solvents for leaf and root plant material were tested earlier, and methanol was demonstrated to be the most efficient solvent, allowing the highest DPPH scavenging activity to be obtained [32,33]. Leaf and root samples of each plant species were homogenised in a ratio of 1:5 with 80% methanol, followed by continuous stirring for 24 h at room temperature using an orbital shaker at 270 rpm. The crude extract was clarified by centrifugation at 5000× *g* for 15 min (TD6 Benchtop Centrifuge Drawell, Chongqing, China). For the determination of antimicrobial activity, leaf and root extracts obtained using 80% methanol were rotary evaporated at 50 °C using DW-ORE2000 Rotary evaporator (Drawell, Chongqing, China), and the residue was re-dissolved in 80% DMSO, sterilised through a Millipore filter (0.22 µm), and then loaded on sterile filter paper disks.

### 2.4. DPPH Radical Scavenging Activity Assay

The DPPH radical scavenging activity of plant extracts was measured according to [34]. Plant extracts (200 µL at different concentrations) were mixed with 2.8 mL of a 0.1 M DPPH solution in 99.9% methanol to a final concentration of 10–300 µg/mL. After incubation at room temperature for 30 min in the dark, the absorbance of the resulting solution was measured at 517 nm using a DU-8800 RS spectrophotometer (Drawell, Chongqing, China). The blank was a methanolic dilution of DPPH, and the absorbance was 0.98 ± 0.02.

The scavenging activity was estimated based on the percentage of DPPH radical scavenged according to the following equation:

Scavenging effect (%) = [(control absorbance − sample absorbance)/(control absorbance)] × 100

The concentrations of the plant extracts required for 50% inhibition (IC_50_) were calculated by plotting the inhibition of DPPH (%) versus the concentration of extract, and the data were fit with a straight line (linear regression).

Y = a × X + b,

IC50 = (50 − b)/a

### 2.5. Total Antioxidant Capacity (Phosphomolybdate Assay)

The total antioxidant capacity (TAC) of the plant extracts was determined by the phosphomolybdate method using ascorbic acid as a standard [35]. Plant extracts (200 µL at different concentrations) were mixed with 2.8 mL of reagent solution (0.6 M sulfuric acid, 28 mM sodium phosphate, and 4 mM ammonium molybdate). The tubes were capped and incubated in a water bath at 95 °C for 90 min. Ascorbic acid was used as a standard. After the samples cooled to room temperature, the absorbance of the mixture was measured at 765 nm against a blank using a DU-8800 RS spectrophotometer, and TAC was expressed as mg ascorbic acid equivalent (AAE) per g DW. A typical blank contained 2.8 mL of the reagent solution, and 200 µL of the methanol and was incubated under the same conditions. Ascorbic acid was used as a standard.

### 2.6. Determination of Total Phenolic Content (TPC)

The total phenolic content (TPC) of the plant extracts was determined using Folin–Ciocalteu’s reagent according to [36]. Plant extracts (200 µL at different concentrations) were mixed with 1.5 mL of Folin–Ciocalteu’s phenol reagent, previously diluted 10 times with double-distilled water. The mixture was incubated for 5 min at 25 °C, after 1.5 mL of Na_2_CO_3_ solution (60 g/L) was added to the mixture. The mixture was kept in the dark for 90 min at 23 °C, after which the absorbance was measured at 750 nm using a DU-8800 RS spectrophotometer. The calibration curve for TPC determination was made using gallic acid as a standard. The TPC was expressed as mg of gallic acid equivalents (GAE) per g DW.

### 2.7. Determination of Total Flavonoid Content (TFC)

The total flavonoid content (TFC) of the plant extracts was estimated using a method described by Park et al. [37]. Plant extracts (300 µL at different concentrations) were mixed with 3.4 mL of 30% methanol, 0.15 mL of NaNO_2_ (0.5 M), and 0.15 mL of AlCl_3_.6H_2_O (0.3 M). After 5 min, 1 mL of NaOH (1 M) was added. The solution was mixed well, and the absorbance was measured against the reagent blank at 506 nm using a DU-8800 RS spectrophotometer. The calibration curve for TFC determination was made using rutin as a standard. The total flavonoid content was expressed as mg of rutin equivalents (RE) per g DW.

### 2.8. Extraction of Plant Samples for the Determination of Ascorbate and Glutathione Contents

Plant material (1 g) was homogenised in 2 mL 5% (w/v) metaphosphoric acid and 1 mM EDTA at 4 °C and then centrifuged for 30 min at 15,000× *g*, and the supernatant was collected for analysis.

### 2.9. Determination of Reduced (ASC) and Oxidised (DHA) Ascorbate Content

ASC and DHA were assayed according to the method described earlier [38]. The assay is based on the reduction of Fe^+3^ to Fe^2+^ by ascorbic acid in acidic solution, and Fe^2+^ forms pink colour complexes with bipyridyl, absorbing at 525 nm using a DU-8800 RS spectrophotometer. The reduction of DHA into ASC was performed by incubation the sample with dithiothreitol (DTT). The excess of DTT was removed by the addition of *N*-ethylmaleimide, and the total ACA was determined. The ASC content was calculated using a standard curve, and the amount of DHA was determined as the difference between total ascorbate and ASC.

### 2.10. Determination of Reduced (GSH) and Oxidised (GSSG) Glutathione

Reduced and oxidised glutathione content were determined by a modified Anderson’s method [39]. GSH was oxidised by 5,5′-dithiobis(2-nitrobenzoic acid) (DTNB), followed by the formation of GSSG and TNB (5-thio-2-nitrobenzene). GSSG was reduced to GSH by the action of glutathione reductase and NADPH, and an increase in absorbance was followed for 4 min at 412 nm using a DU-8800 RS spectrophotometer. GSSG was assayed from the sample after removal of GSH by 2-vinylpyridine derivatisations. The total glutathione and GSSG content were calculated using a standard curve. The amount of GSH was determined as the difference between total glutathione and the GSSG.

### 2.11. Determination of Antibacterial Activity

The Kirby–Bauer diffusion method was employed for antibacterial activity screening [40]. The *Escherichia coli* ATCC 25,922 strain was used in the study. The bacteria were grown in LB broth for 16–18 h at 37 °C (10^9^–10^10^ CFU/mL). Sterile blank discs with a 6 mm diameter were individually placed on an LB nutrient agar plate covered with 300 µL of the bacterial strain. Different concentrations of plant leaf or root extracts (25, 50, and 100 µg/disk) were applied to the disc. These plates were incubated at 37 °C for 24 h. The antimicrobial activity was determined in triplicate by measuring the diameter of the inhibition zone (IZ, mm). Amoxicillin (30 µg/disk) was used as the positive control. Dimethyl sulfoxide (80%) was used as a negative control.

### 2.12. Determination of Minimal Inhibitory Concentration (MIC)

The antibacterial activity of the plant extracts was determined using sterile 3.5 mL 24-well plates [41]. The 12 wells of each row were filled with 1.5 mL of sterilised LB broth. Sequentially, wells 2–11 received an additional 1.5 mL of a mixture of culture medium and plant extract serially diluted to create a concentration sequence from 0.5 mg/mL to 15 mg/mL. Well 1 was the growth control, and well 12 was the antibiotic control (amoxicillin, 0.1 mg/mL). The plates were incubated for 24 h at 37 °C. The resulting turbidity was observed, and after 24 h, MIC was determined to be where growth was no longer visible by the assessment of turbidity by optical density readings at 600 nm using a DU-8800 RS spectrophotometer. At least three repetitions were run for each assay. According to Bussmann et al. [42] strong antibacterial activity was defined as MIC < 5 mg/mL.

### 2.13. Statistical Analysis

A variety of descriptive and inferential statistical methods were used for statistical analysis of the data. For all assays, at least four biological replicates were used, each with three technical replicates, and the data obtained were expressed as arithmetical means ± standard deviation. The results for each analysed parameter were compared between all plant samples using an ANOVA F test. Initially, we checked whether the data met the requirements of parametric statistical criteria: the assumption of normal distribution and homogeneity of variances. Kolmogorov–Smirnov and Shapiro–Wilk tests were used to check whether our data were normally distributed. Homogeneity of variances was tested using Levene’s test. Both requirements were met for our dataset. The significance of the differences between mean values was evaluated using Tukey’s HSD test. Then, we compare each parameter between leaves and roots for each plant using an independent sample *t*-test. There was a statistically significant difference at the 0.05 significance level.

To evaluate significant relationships between experimental parameters, correlation and regression analysis methods were used. 

### 2.14. Molecular Docking

#### 2.14.1. Ligand Preparation

Ascorbate is the predominant chemical species at physiological pH [43]. On the other hand, GSH exists as a zwitterion under the same conditions, with the glutamate moiety carrying both a positively charged NH_3_^+^ group and a negatively charged COO^−^ group, while the glycine moiety has a COO^−^ group [44]. To perform the docking simulations, we downloaded the 3D structure for sodium ascorbate (PubChem CID: 23667548) as an SDF file from PubChem. The file was then edited in Discovery Studio Biovia v21.1.0.20298 and saved as PDB file for use as a ligand in the molecular docking simulation. The GSH structure for docking was prepared through a series of steps. Initially, the SMILES string was obtained from PubChem (PubChem CID: 92043348). This string was then submitted to the ProNovo online server (https://novoprolabs.com/tools/smiles2pdb, accessed on 9 April 2025), which utilises the Smiles2pdb tool to generate the 3D structure. The resulting PDB file was further edited in Discovery Studio Biovia, where hydrogen atoms were added, charges were adjusted, and energy minimisation was carried out using the CHARMm force field.

#### 2.14.2. HDOCK Docking

Subsequently, the structures (PDB file) were uploaded to the HDOCK software for molecular docking. In particular, the dockings were performed against OmpX (PDB ID: 1QJ8) using the blind docking modality and standard parameters in HDOCK [45]. The program calculates an energy score, referred to as the HDOCK score, which is dimensionless. Lower values of the HDOCK score indicate a stronger affinity between the ligands and the target. More details about the HDOCK software can be found at http://hdock.phys.hust.edu.cn (accessed on 9 April 2025).

## 3. Results and Discussion

### 3.1. Antioxidant Activity and Total Antioxidant Capacity

**Figure 1:**
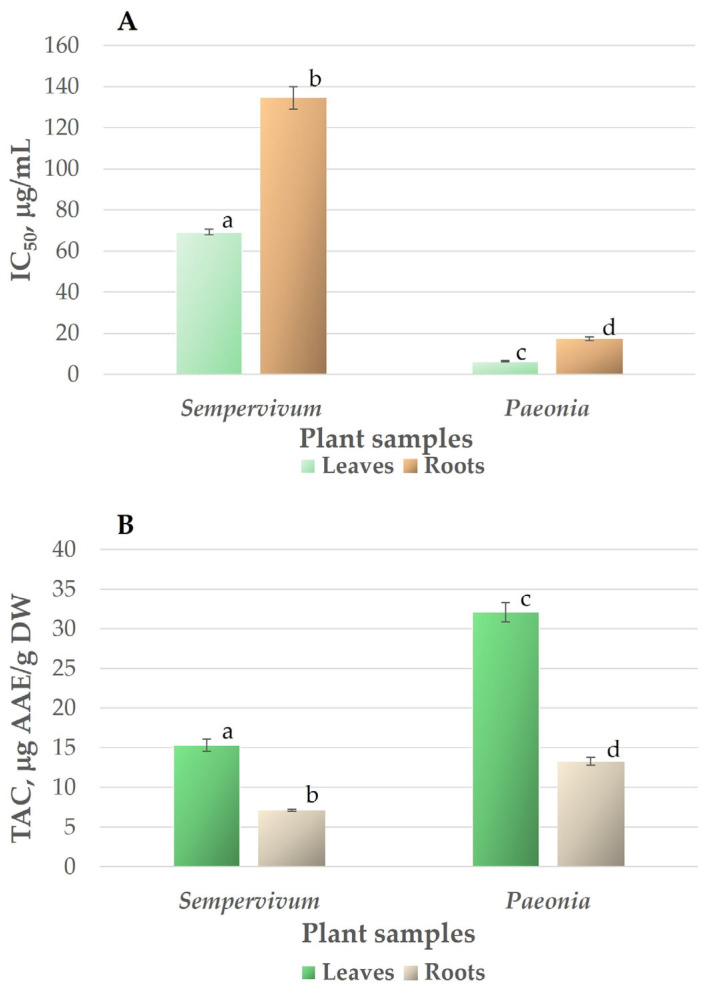
Antioxidant activity of *Sempervivum transcaucasicum* and *Paeonia daurica* subsp. *mlokosewitschii* leaf and root extracts determined by DPPH method (**A**) and total antioxidant capacity (**B**). The data are presented as means ± SD (bars), n = 3. Different letters indicate a significant difference between values of the determined parameter for leaf and root samples of both plants using ANOVA F test, followed by Tukey’s HSD test at *p* < 0.05.

The interest in antioxidants among scientists in medical professionals is continuously increasing due to their protective roles against oxidative deterioration and in the body against oxidative-stress-mediated pathological processes [46]. The scavenging ability of DPPH free radicals and total antioxidant capacity are widely used to analyse the antioxidant potential of plants. The radical scavenging activity measured using the DPPH method is shown in Figure 1A. The IC_50_ value, determined graphically, was calculated using the linear regression equation derived from the calibration curve. A low IC_50_ value indicates high antioxidant activity. The antioxidant activity of leaf and root extracts of *Sempervivum transcaucasicum* (IC_50_ were 69.24 and 134.53 µg/mL, respectively) was relatively low compared to ascorbic acid (IC_50_ was 13.05 ± 0.23 µg/mL). Significantly higher radical scavenging activity was revealed for the leaf and root extracts of *Paeonia daurica* subsp. *mlokosewitschii* (IC_50_ of leaf extract was 6.31 µg/mL), and the IC_50_ for root extract was 17.34 µg/mL, which was 11- and 8-fold higher than for the respective *Sempervivum transcaucasicum* extracts. Since the data on antioxidant activity and the content of antioxidant compounds for the species investigated in our study are not available in the literature, we compared the obtained data with the results for different species of the same genus. The IC_50_ for *Sempervivum transcaucasicum* leaf extract determined in this study was in the range demonstrated earlier for leaf extracts of *Sempervivum tectorum* L. (IC_50_ of 34.9–78.6 µg/mL, depending on the extractant used) [47]. The results obtained for *Paeonia daurica* subsp. *mlokosewitschii* were similar to those obtained for *Paeonia officinalis* L., where higher antioxidant activity of leaf extracts in comparison with root extracts was demonstrated by DPPH assay [48]. The antioxidant activity of the leaf extract of *Paeonia daurica* subsp. *mlokosewitschii* demonstrated in this study was several times higher than that of *Paeonia daurica* subsp. *daurica* Andrews (IC_50_ of 46–51 µg/mL) [49] and was comparable to the antioxidant activity shown for 13 *Paeonia lactiflora* Pall. cultivars (IC_50_ of 3–25 µg/mL) [50]. The results of our study indicate that the antioxidant activity of the root extract of *Paeonia daurica* subsp. *mlokosewitschii* was comparable with the activity of *Paeonia daurica ssp. macrophylla* (Albov) D.Y. Hong root extracts (IC_50_ of 16.55–73.91 µg/mL for fractions obtained using different extractants) [51].

In our study, total antioxidant capacity (TAC) was also determined [35] in addition to determining antioxidant activity by the DPPH method. The reason for the use of two methods was the fact that the determination of antioxidant capacity, especially for phenolic-rich samples, depends on the choice of assay [52], and therefore, the antioxidant activity should not be concluded based on a single method [46]. It was shown that the DPPH assay is well correlated with the content of phenolic compounds, whereas the TAC method is non-selective for phenolic compounds, but efficient for a wide range of samples exhibiting antioxidant activity [53].

Total antioxidant capacity was also higher in *Paeonia daurica* subsp. *mlokosewitschii* leaf and root extracts in comparison with *Sempervivum transcaucasicum* extracts (Figure 1B). A significant difference among the plant extracts was observed for the TAC assay (*p* < 0.05), revealing lower values for *Sempervivum transcaucasicum* leaf and root extracts (15.32 and 7.13 µg AAE/g DW (ascorbic acid equivalent), respectively), whereas the corresponding indexes were twice as high for *Paeonia daurica* subsp. *mlokosewitschii* leaf and root extracts (32.10 and 13.27 µg AAE/g DW, respectively). Data on the TAC of *Paeonia daurica* subsp. *mlokosewitschii* and *Sempervivum transcaucasicum* are not available in the literature. Antioxidant activity depends on the presence of bioactive compounds with antioxidant properties, and the concentration of these compounds present in the extract is important in showing antioxidant activity [54]. Therefore, during the next stage of our study, we determined the content of major antioxidants, known to contribute to the antioxidant activity of plant extracts, such as polyphenols, flavonoids, and water-soluble antioxidants.

### 3.2. Total Phenolic and Flavonoid Content

One of the main groups of compounds with antioxidant activity in plants is phenols, possessing one or more aromatic rings with one or more hydroxyl groups [55]. The structure of phenols allows the stabilisation and relocation of the unpaired electrons, thus facilitating the donation of hydrogen atoms and electrons from their hydroxyl groups [56]. The total phenolic content varies depending on the plant species, tissue, developmental stage, habitat types, soil composition, climatic factors, and altitude [57].

**Figure 2:**
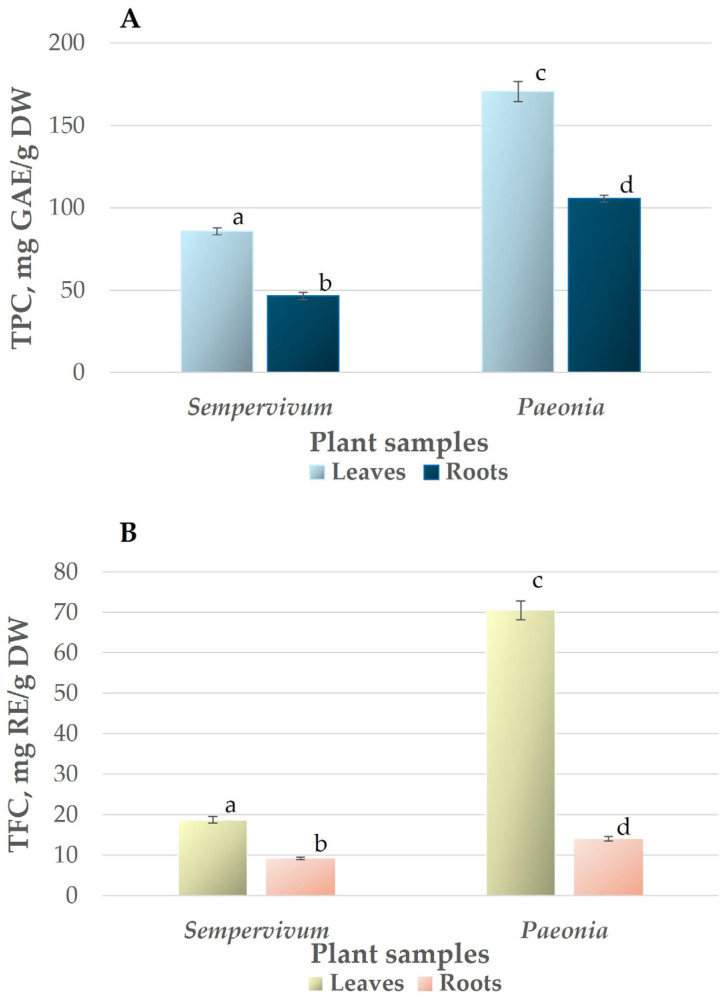
Phenol (**A**) and flavonoid contents (**B**) in leaves and roots of *Sempervivum transcaucasicum* and *Paeonia daurica* subsp. *mlokosewitschii*. The data are presented as means ± SD (bars), n = 3. Different letters indicate a significant difference between values of the determined parameter for leaf and root samples of both plants using ANOVA F test, followed by Tukey’s HSD test at *p* < 0.05.

The total phenolic content (TPC) measured using gallic acid equivalent (GAE) as a standard reference was twice as high in *Paeonia daurica* subsp. *mlokosewitschii* leaf and root extracts (170.58 and 105.61 mg GAE/g DW, respectively) in comparison with *Sempervivum transcaucasicum* leaf and root extracts (85.38 and 46.52 mg GAE/g DW) (Figure 2A). The results obtained for *Sempervivum transcaucasicum* leaf extract are similar to those demonstrated for *Sempervivum marmoreum* L. leaf extracts (40.5–85.9 mg GAE/g DW, depending on extraction method) [58] and *Sempervivum davisii* Muirhead (TPC = 133.6 mg GAE/g DW) [22]. The TPC in *Paeonia daurica* subsp. *mlokosewitschii* leaf and root extract was higher than TPC obtained for several other *Paeonia* species. Thus, lower TPC was demonstrated for the leaf extracts of various hybrids of *Paeonia suffruticosa*, *Paeonia potaninii*, *Paeonia ludlowii* (48.3–98.7 mg GAE/g FW) [59], and 13 *Paeonia lactiflora* Pall. cultivars (55.95–105.33 mgGAE/g DW) [50]. Lower TPC was also demonstrated for root extracts of 15 species and 2 subspecies of wild Paeony (4.40–6.56 mg GAE/g DW) [60]. The results for *Paeonia daurica* subsp. *mlokosewitschii* leaf extract obtained in this study were comparable to data for two *Paeonia* species: *Paeonia officinalis* L. (TPC of 187.2–285.9 mg GAE/g DW for leaf extracts and 41.4–59.1 mg GAE/g DW for root extract) [48] and *Paeonia ostii* T. Hong & J.X. Zhangin (TPC of 280.38 mg GAE/g DW for leaf extracts and 125.48 mg GAE/g DW for root extract) [61].

Flavonoids are a class of secondary plant phenolics with significant antioxidant and chelating properties [62]. Studies on flavonoids have shown a wide range of antioxidant, antibacterial, antiviral, anti-inflammatory, anticancer, and anti-allergic activities (reviewed in [13,14]). A pattern like TPC was revealed for TFC: the content was 3.8 and 1.5 times higher in *Paeonia daurica* subsp. *mlokosewitschii* leaf and root extracts (70.51 and 14.05 mg RE/g DW) in comparison with *Sempervivum transcaucasicum* leaf and root extracts (18.78 and 9.23 mg RE/g DW, respectively) (Figure 2B). The TFC for *Paeonia daurica* subsp. *mlokosewitschii* leaf extracts was higher than the content shown for 13 *Paeonia lactiflora* Pall. cultivars (10.52–23.26 mg RE/g DW) [50], while for root extracts, the values obtained in this study were in the range shown for 15 species and 2 subspecies of wild *Paeony* (3.52–11.57 CE mg/100 g DW) [60]. For *Sempervivum transcaucasicum* leaf extracts, results were in the range shown for leaves of another *Sempervivum* species: *Sempervivum marmoreum* L. (TFC in the range of 12.7–19.3 mg RE/g DW, depending on extraction method) [58].

### 3.3. Ascorbate and Glutathione Content

During the next stage of our study, we determined the content of the most abundant low molecular weight antioxidants in plant cells, ascorbate and glutathione, whose primary functions are related to interactions with ROS [63].

ASC is a potent antioxidant, mostly abundant in leaves, where it is present in higher concentrations compared to GSH [64]. ASC can directly scavenge OH, O_2_^•−^, and ^1^O_2_ and reduce H_2_O_2_ into water via the ascorbate peroxidase reaction [65]. As a result of this reaction, monodehydroascorbate, which can be spontaneously converted to dehydroascorbate (DHA), is formed. Unlike ASC, DHA lacks antioxidative capability and is converted back to ASC by the addition of two electrons from GSH by DHA reductase [66]. Therefore, the ASC/DHA ratio is an important indicator of the redox status of the cell [67].

The ASC content was several-fold higher in the leaves than in the roots of both species (Table 1). The highest ASC content was revealed in *Paeonia daurica* subsp. *mlokosewitschii* leaves, which was 4.6 times higher than the content in *Sempervivum transcaucasicum* leaves. The same pattern was revealed in root samples, where the ASC content in *Paeonia daurica* subsp. *mlokosewitschii* exceeded that of *Sempervivum transcaucasicum* by 3.5 times. Similarly to the results of our study, a low amount of ASC (less than 1 mg/g DW) was demonstrated for *Sempervivum tectorum* L. leaves [20]. The results obtained for our study for *Paeonia daurica* subsp. *mlokosewitschii* were 2–3 times higher than the results obtained for *Paeonia obovata* Maxim. and *Paeonia oreogeton* S. Moore (ASC content of 151.8–155.2 mg/100 g DW in leaf extracts and 30.5–31.5 mg/100 g DW in root extracts) grown in the Forest–Steppe Zone of Western Siberia [68]. The content of DHA was significantly lower in *Paeonia daurica* subsp. *mlokosewitschii* leaves in comparison with *Sempervivum transcaucasicum;* however, in *Paeonia* roots, it was higher. Despite this finding, the ASC/DHA ratio was significantly higher in *Paeonia daurica* subsp. *mlokosewitschii* leaves and roots. The ASC/DHA ratio indicates the ability for ROS scavenging and reflects the redox status of the glutathione pool [67].

Due to its importance in redox and regulatory functions, GSH is the most abundant and best described low-molecular-weight thiol in plants [69]. GSH reacts with superoxide and H_2_O_2_, but this reaction is relatively slow compared with ASC [63]. Its main role as an antioxidant is determined by its ability to regenerate ASC either enzymatically via the ASC-GSH cycle or non-enzymatically [64]. The GSH content was significantly higher in the leaves than in the roots of both species (Table 2). The highest GSH content was revealed in *Paeonia daurica* subsp. *mlokosewitschii* leaves and roots, and it was 10 times higher than in the corresponding organs of *Sempervivum transcaucasicum*. The GSH redox potential depends on the GSH concentration and the GSH/GSSG ratio [70]; therefore, high GSH/GSSG ratios, revealed for *Paeonia daurica* subsp. *mlokosewitschii* leaves and roots indicate high antioxidant potential, providing the plant with the ability to fast recycle GSH.

The TPC, TFC, ASC, and GSH contents of both leaf and root extracts of both species showed a significant and strong positive correlation (*p* < 0.001) with DPPH-scavenging ability and TAC. The r^2^ values between TPC, TFC, ASC, and GSH contents and DPPH-scavenging ability and TAC for leaves and roots of both species were in the range of 0.91–0.99. These findings are in agreement with previously published data [71], indicating that phenolic compounds of the plant extracts may be one of the major contributors to the antioxidant activity in ROS neutralisation. The results obtained also confirm the major role of ASC and GSH in the total antioxidant activity of the plant samples.

Accordingly to our previous study of the effect of different extractants on antioxidant activity determined by DPPH radical scavenging activity assay [33], the antioxidant activity of methanol extracts is higher than the activity of extracts obtained using ethanol, commonly used for the extraction of these plants in traditional Georgian medicine. Therefore, due to higher extraction efficiency, the methanol extraction followed by the evaporation of methanol can be recommended for the extraction of these plant species. However, while methanol enables more efficient extraction of certain antioxidant compounds, variations in extractant polarity can significantly influence the phytochemical profile and, consequently, the spectrum and intensity of biological effects. This underscores the importance of future studies that prepare and evaluate extracts using traditional methods employed by local populations.

### 3.4. Antibacterial Activity of Plant Extracts

**Figure 3:**
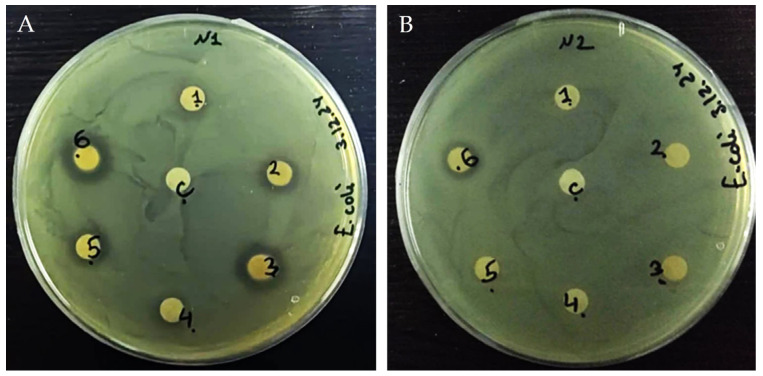
Zone of inhibition of plant extracts against *Escherichia* assessed by the disc-diffusion method. Paper disc diameter, 6 mm. The concentrations of all sample extracts were 200 mg/mL, and different aliquots were taken for testing the indicated amounts of plant extracts. (**A**). *Paeonia daurica* subsp. *mlokosewitschii*. 1–3 leaf extract (25; 50, and 100 µg/disk), 4–6 root extract (25; 50 and 100 µg/disk); (**B**). *Sempervivum transcaucasicum*. 1–3 leaf extract (25; 50, and 100 µg/disk), 4–6 root extract (25; 50 and 100 µg/disk).

The antibacterial activities of *Sempervivum transcaucasicum* and *Paeonia daurica* subsp. *mlokosewitschii* extracts were first tested by agar disc diffusion tests (Figure 3 and Table 3). Analysis of the inhibition zone results showed that *Sempervivum transcaucasicum* leaf extract did not possess an antibacterial effect against *E. coli* in a concentration range of 25–100 µg/disk, and a weak antibacterial effect was revealed for *Sempervivum transcaucasicum* root extract only for the concentration of 100 µg/disk. Both leaf and root extracts of *Paeonia daurica* subsp. *mlokosewitschii* had an antibacterial effect against *E. coli* in the whole range of the applied concentration, and the effect was concentration dependent. The significant differences between the diameter of the inhibition zone (IZ) among *Paeonia daurica* subsp. *mlokosewitschii* leaf and root extracts were not revealed. For the determination of the effective concentrations, a broth microdilution test was used (Table 3). The highest MIC was determined for *Sempervivum transcaucasicum* root extract (5 mg/mL). The strong antibacterial activity was revealed for the leaf and root extracts of *Paeonia daurica* subsp. *mlokosewitschii.* The lowest MIC of 2 mg/mL was revealed for *Paeonia daurica* subsp. *mlokosewitschii* leaf extract, followed by MIC for *Paeonia daurica* subsp. *mlokosewitschii* root extract (3 mg/mL). The antibacterial effect of ethanol and aqueous *Sempervivum tectorum* L. leaf extracts was demonstrated against multiple antibiotic-resistant *E. coli* ATCC 25922, notably, the strongest effect was observed with the aqueous extracts [72]. The antibacterial activity against *E. coli* was demonstrated for the whole-plant extracts of *Paeonia emodi* Wall. ex Royle [73], as well as *Paeonia wendelboi* Rukšāns & Zetterl leaf and root extracts [74]. Volatile constituents, isolated from roots of Greek Paeonia taxa (*Paeonia clusii* Stern subsp. *clusii*, and *Paeonia parnassica* Tzanoud.), exhibited antibacterial activity against *E. coli* (MIC values of 2.23 and 4.23 mg/mL) [75].

The antibacterial *Paeonia daurica* subsp. *Mlokosewitschii* leaf and root extracts would be linked to the presence of various classes of secondary metabolites. Phenols and flavonoids are classes of secondary metabolites known to have antiplasmodial and antibacterial activities [76]. Phenolic compounds act on bacteria by modifying the permeability of cell membranes, inducing changes in various intracellular functions by binding of the phenolic compounds to enzymes, or via the modification of the cell wall rigidity due to different interactions with the cell membrane [77]. The antibacterial activity of flavonoids is attributed to three mechanisms: cytoplasmic membrane damage; inhibition of topoisomerase; and, as a consequence, the inhibition of nucleic acid synthesis and NADH-cytochrome *c* reductase inhibition, leading to the inhibition of the energy metabolism [77]. Based on the results obtained in this study, further analysis of individual phenolic and flavonoid compounds will be performed. The identification of major phenolic and flavonoid compounds from *Paeonia daurica* subsp. *Mlokosewitschii* will allow the determination of their potential health benefits and assess the possibility of application of such compounds in various fields like food science, pharmaceuticals, and natural product chemistry. Also, the identification of the structure of these compounds will allow us to perform docking studies, evaluating the antimicrobial activity of such components.

ASC has a long and controversial history of reported antimicrobial capabilities [78]. The most common and simplistic explanation of the antibacterial effect is the low pH created by ASC. A bactericidal effect of ASC against *Pseudomonas aeruginosa*, *Mycobacterium tuberculosis*, *Staphylococcus aureus*, *Enterococcus faecalis*, and *Acinetobacter baumannii* was demonstrated [78]. The MIC of ASC of 34 mM was reported for *Escherichia coli* [79]. The effective ASC concentration that could inhibit the growth of the majority of 100 non-repeated *E. coli* clinical isolates from patients with urinary tract infection was 1.25 mg/mL, and an excellent anti-biofilm effect of ASC against these isolates was also demonstrated [80]. Therefore, the antibacterial activity revealed for *Paeonia daurica* subsp. *Mlokosewitschii* leaf, and root extracts can be associated with the high content of ASC.

The clinical relevance and molecular details of the antibacterial activity of GSH are currently unclear. Antibacterial activity of exogenous GSH in *Acinetobacter baumannii* [81] and *Pseudomonas aeruginosa* [82], as well as synergism on antibiotics, were reported. However, the mechanism of GSH action as an antibacterial agent remains unknown.

### 3.5. Computational Investigation of OmpX as a Molecular Target for Plant-Derived Antioxidants in E. coli

**Figure 4:**
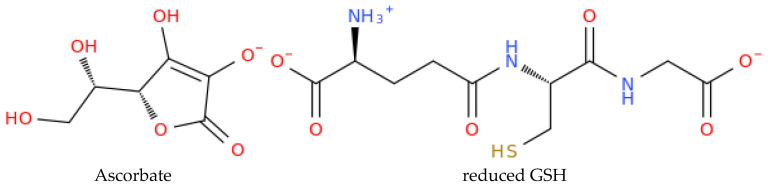
Structures of the ionic forms of the ligands investigated in the molecular docking.

Given that the water-soluble components of the most active *Paeonia daurica* subsp. *Mlokosewitschii* extracts are likely responsible for the first attack on *E. coli*, considering the disc diffusion method employed, we decided to explore in silico the binding of reduced glutathione and ascorbic acid to one of the biologically relevant external targets on the bacterial membrane, the Outer Membrane Protein (Omp) family, and more specifically OmpX (PDB ID: 1QJ8), which is implicated in *E. coli* adhesion, invasion, and immune evasion (Figure 4).

**Figure 5:**
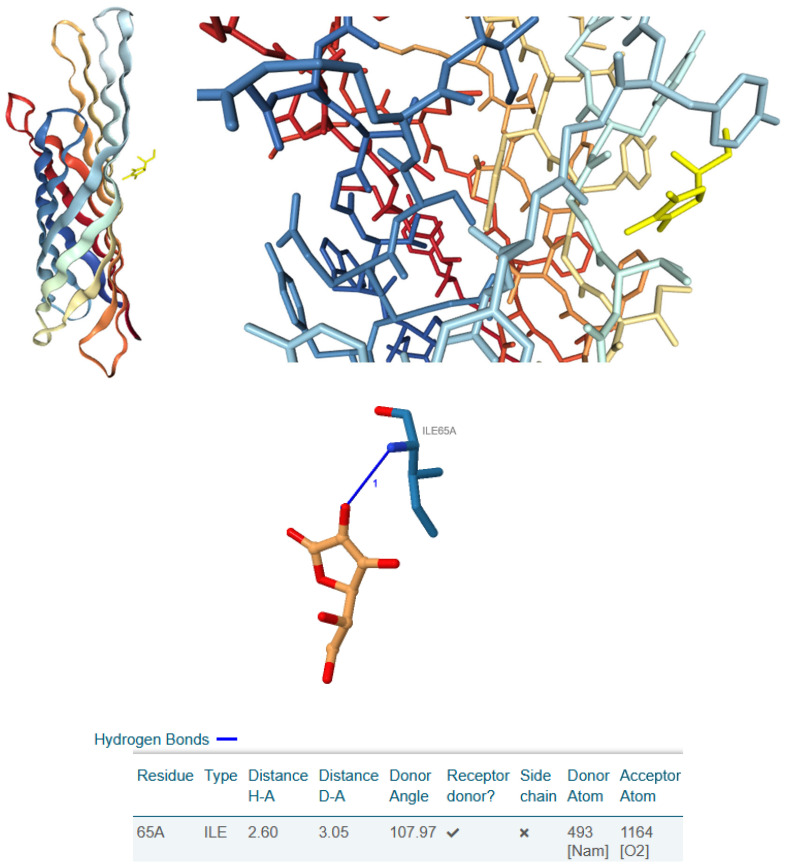
The figure displays the pose view of the best-ranked (top 1) docking result from HDOCK molecular docking between OmpX and ASC. The visualisation includes an overall view of the complex and a zoomed-in view of the binding centre. At the bottom, the PLIP-generated 2D interaction diagram illustrates the specific ligand–protein interactions. The corresponding interaction data, such as bond length and residue details, are also annotated.

**Figure 6:**
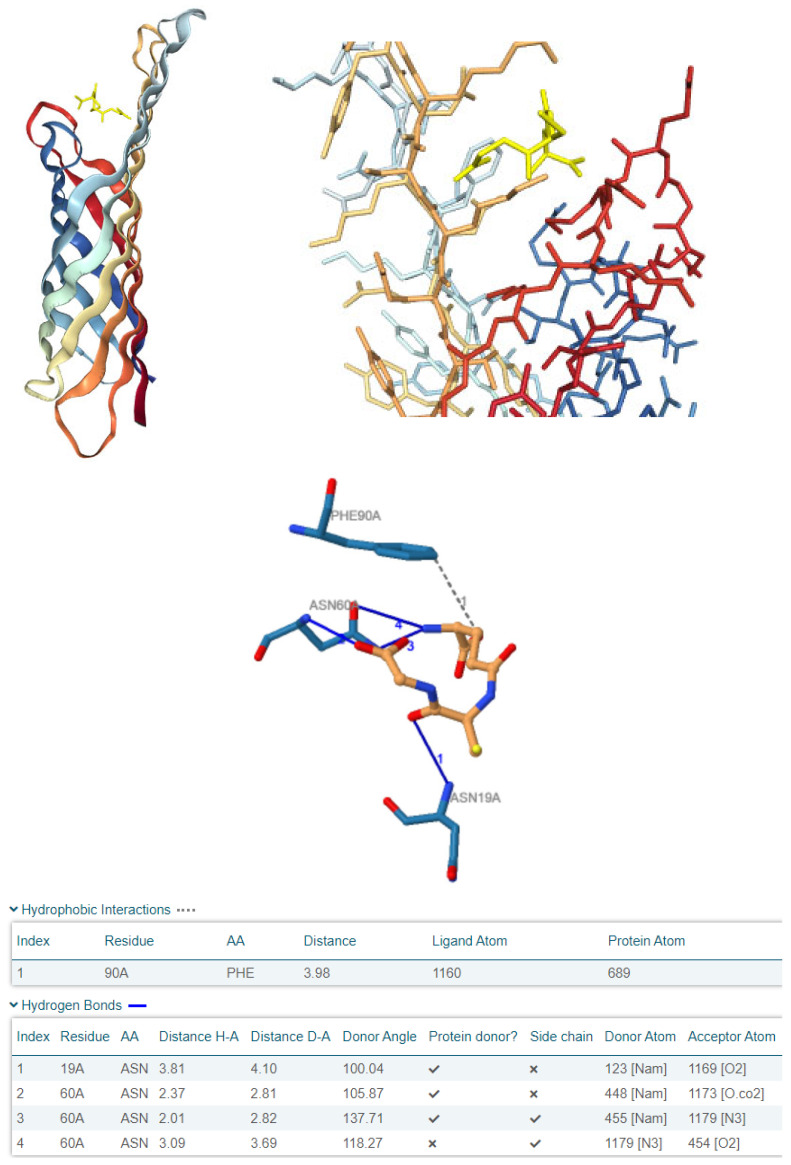
The figure illustrates the pose view of the best-ranked docking result from HDOCK molecular docking for the complex between OmpX and the ionic form of GSH. The visualisation includes an overall view of the complex and a zoomed-in view of the binding centre. At the bottom, the PLIP-generated 2D interaction diagram depicts the specific ligand–protein interactions, including hydrophobic contacts and hydrogen bonds. The corresponding interaction data, such as bond lengths and residue details, are also annotated.

The selection of these ligands for molecular docking was based on their high abundance as low-molecular-weight constituents in the active plant extracts, their documented antibacterial activity against clinically relevant pathogens, and their likely role in the initial interaction with bacterial cells. Given that the antimicrobial activity assessment was performed using a disc diffusion method, which emphasises water-soluble fractions, we postulated that these components would have a significant role in the observed antibacterial effects. Additionally, we assume that when antibacterial compounds engage with bacteria, they first interact with the bacterial outer membrane. Since *Escherichia coli* serves as a model organism for Gram-negative bacteria, we aimed to investigate whether ASC and GSH could bind in silico to the outer membrane protein OmpX. OmpX, as an outer membrane protein, plays a critical role in bacterial adhesion, immune evasion, biofilm formation, and overall structural integrity of the outer membrane. The loops of OmpX are particularly important as they protrude into the extracellular environment and are involved in interactions with external molecules, the bacterial outer membrane’s integrity, contributing to their antibacterial activity [83]. Interestingly, ASC demonstrated a tendency to externally bind to OmpX (Figure 5), whereas GSH was predicted to have the highest affinity, binding to one of OmpX’s openings, specifically in the loop-rich region (Figure 6). These findings suggest that OmpX, a small integral outer membrane protein involved in bacterial adhesion, immune evasion, and biofilm formation, could serve as a potential target for disrupting bacterial functionality by *Paeonia daurica* subsp. *mlokosewitschii* leaf and root extracts.

As for the binding affinities, both ligands were found to exhibit favourable interactions, as evidenced by their negative HDOCK scores (Table 4). As illustrated in Table 4, GSH exhibited a higher binding affinity for OmpX compared to ASC, as demonstrated by its superior docking scores (Top-1 Pose Score: −110.66 vs. −95.86). Furthermore, GSH formed multiple interactions with OmpX, including hydrophobic contacts with Phe90 and several hydrogen bonds with Asn19 and Asn60, whereas ASC established only one hydrogen bond with Ile65. These findings highlight the stronger and more complex interaction dynamics of GSH with OmpX relative to ASC.

Due to its higher predicted binding affinity and its distinctive tendency to interact from the loop side of OmpX, GSH has emerged as a more compelling candidate. Its ability to form multiple interactions, including hydrophobic and hydrogen bonds with key residues such as Phe90, Asn19, and Asn60 (Table 4), further supports its potential to disrupt OmpX functionality. This positions GSH as an intriguing molecule for modulating bacterial outer membrane proteins like OmpX, with promising implications for antibacterial strategies. The extracts of *Paeonia daurica* subsp. *mlokosewitschii,* possessing high antioxidant and antimicrobial activities, can be used for the prevention or treatment of conditions associated with oxidative stress and a greater susceptibility to microbial infections, including those induced by xenobiotics. Thus, it is known that oxidative stress is the major driving mechanism of chronic respiratory disease, associated with environmental exposures to xenobiotics such as air pollution and nutrition [84], and antioxidant therapy was suggested for the treatment of this group of diseases. Several molecular mechanisms underlying the progression of sepsis are associated with increased formation of ROS and exhausted antioxidant pathways [85]. Inflammatory cytokines released during sepsis affect the expression of xenobiotic receptors and drug-metabolising enzymes [86]. Therefore, the application of antioxidants with antimicrobial properties in addition to primary therapy could improve the outcomes of patients with sepsis. Remarkably, recent clinical evidence supports the link between oxidative stress, immune dysregulation, and microbial susceptibility in inflammatory conditions such as septic shock, where antioxidant co-therapy significantly reduced cytokine levels and oxidative stress markers, highlighting the therapeutic relevance of antioxidant and antimicrobial plant compounds as observed in our study [87].

## 4. Conclusions

The comparison of two Georgian medicinal plants, representatives of different families, *Sempervivum transcaucasicum* (Crassulaceae) and *Paeonia daurica* subsp. *mlokosewitschii* (Paeoniaceae), revealed that both leaf and root extracts of *Paeonia daurica* subsp. *mlokosewitschii* exhibit high DPPH radical scavenging activity and total antioxidant capacity.

The methanolic leaf and root extracts of *Paeonia daurica* subsp. *mlokosewitschii* displayed pronounced antibacterial activity against *Escherichia coli*, with the lowest MIC values being obtained for the *Paeonia daurica* subsp. *mlokosewitschii* leaf extract. Our in silico study offers a mechanistic hypothesis for the observed antibacterial activity of *Paeonia daurica* extracts. By targeting the loop region of OmpX, reduced glutathione might act as an inhibitor, disrupting key bacterial functions and reducing *E. coli*’s survival and virulence. Overall, this emphasises the role of water-soluble plant-derived components as promising antibacterial agents with the potential to target essential bacterial structures like OmpX, thereby supporting further exploration in antimicrobial research. According to our study, *Paeonia daurica* subsp. *mlokosewitschii* is a valuable natural source of phenolic and flavonoid compounds as well as water-soluble low-molecular-weight antioxidants, which can decrease the impact of ROS produced during various diseases.

The results of the study will be used for the identification of biologically active compounds from *Paeonia daurica* subsp. *mlokosewitschii* and the exploration of possibilities to use this species as a substitute for antioxidant and antibacterial medications, as well as the development of food supplements. In perspective, the antioxidant properties of *Paeonia daurica* subsp. *mlokosewitschii* suggest its potential role in mitigating xenobiotic-induced oxidative damage, highlighting its promise as a natural protective agent against environmental and chemical stressors and pointing to the need for further biological investigations.

## Figures and Tables

**Table 1:** The content of reduced (ASC) and oxidised (DHA) ascorbate and the ASC/DHA ratio in the leaves and roots of *Sempervivum transcaucasicum* and *Paeonia daurica* subsp. *mlokosewitschii*.

Plant/Organ	Reduced and Oxidised Ascorbate Content		
	ASC, mg/100 g DW	DHA, mg/100 g DW	ASC/DHA
**Sempervivum**			
Leaves	78.36 ± 4.80 a	63.39 ± 6.45 a	1.24 ± 0.11 a
Roots	33.70 ± 2.09 b	29.99 ± 2.54 b	1.26± 0.14 a
**Paeonia**			
Leaves	359.12 ± 12.80 c	46.96 ± 3.47 c	7.696 ± 0.76 b
Roots	117.27 ± 6.97 d	52.93 ± 3.65 d	2.23 ± 0.0.22 c

The data are presented as means ± SD, n = 3. Different letters indicate a significant difference between values of the determined parameter for leaf and root samples of both plants using ANOVA F test, followed by Tukey’s HSD test at *p* < 0.05.

**Table 2:** The content of reduced (GSH) and oxidised (GSSG) glutathione and the GSH/GSSG ratio in the leaves and roots of *Sempervivum transcaucasicum* and *Paeonia daurica* subsp. *mlokosewitschii*.

Plant/Organ	Reduced and Oxidised Glutathione Content		
	GSH, mg/100 g DW	GSSG, mg/100 g DW	GSH/GSSG
***Sempervivum***			
Leaves	3.00 ± 0.21 a	1.58 ± 0.13 a	1.92 ± 0.20 a
Roots	2.16 ± 0.20 b	0.35 ± 0.02 b	6.19 ± 0.43 b
***Paeonia***			
Leaves	29.95 ± 2.92 c	2.78 ± 0.27 c	10.84 ± 1.18 c
Roots	23.56 ± 1.63 d	1.74 ± 0.07 d	13.61 ± 1.28 d

The data are presented as means ± SD, n = 3. Different letters indicate a significant difference between values of the determined parameter for leaf and root samples of both plants using ANOVA F test, followed by Tukey’s HSD test at *p* < 0.05.

**Table 3:** Inhibition zone and minimal inhibitory concentration (MIC) of *Sempervivum transcaucasicum* and *Paeonia daurica* subsp. *mlokosewitschii* leaf and root extracts. Amoxicillin (30 µg/disk) was used as a control.

Plant Species/Organ and Control (Amoxicillin)	Diameter of Inhibition Zone, mm at 100 µg/disk	MIC, mg/mL
***Sempervivum transcaucasicum***		
Leaves	NI	NI
Roots	8.8 ± 0.3 a	5
***Paeonia daurica*** ** subsp. *mlokosewitschii***		
Leaves	13.3 ± 0.3 b	2
Roots	13.1 ± 0.5 b	3
Amoxicillin	27.8 ± 0.8 c	0.004

The data are presented as means ± SD, n = 3. Different letters indicate a significant difference between values of the determined parameter for leaf and root samples of both plants using ANOVA F test, followed by Tukey’s HSD test at *p* < 0.05. NI—no inhibition.

**Table 4:** Docking analysis of GSH and ASC with OmpX: metrics and interaction summary.

	GSH	ASC
**Top-1 pose score**	−110.66	−95.86
**Average score (top 1–3)**	−108.65	−91.54
**SD of top 1–3 scores**	1.76	3.46
**OmpX interactions**	Hydrophobic interaction with Phe90 (distance: 3.98 Å); H bond with Asn19 (H-A distance: 3.80 Å); multiple H bonds with Asn60 (H-A distances: 2.37 Å, 2.01 Å, 3.09 Å)	H bond with Ile65 (H-A distance: 2.60 Å)

## Data Availability

The original contributions presented in this study are included in the article. Further inquiries can be directed to the corresponding authors.

## Abbreviations

The following abbreviations are used in this manuscript:

ASC	Reduced ascorbate
DHA	Oxidised ascorbate
GSH	Reduced glutathione
GSSG	Oxidised glutathione
IZ	Inhibition zone
MIC	Minimum inhibitory concentration
OmpX	Outer Membrane Protein X
PDB	Protein Data Bank
SD	Standard deviation
TFC	Total flavonoid content
TPC	Total phenolic content
